# Mr. Lawrence on Dislocations of the Vertebræ

**Published:** 1828-04-01

**Authors:** 


### *On Dislocations of the Vertebræ.* By W. LAWRENCE, F. R. S.

[Med. Chir. Trans. Vol. XIII.]

SOME of our best surgical authorities maintain that, with the exception of the first and second bones of the neck, complete dislocations of the vertebræ, without fracture, are nearly impossible. Boyer, Delpech, Sir Astley Cooper, may be cited on this side of the question. Other surgeons (and even physicians) have affirmed, that the said bones may be luxated. RUST says, that even the lumbar and dorsal vertebræ may be dislocated—and German research has pointed out several recorded cases of this kind—but, whether true or false, is another question. One case Rust mentions as having been treated by himself. "The injury was produced by a severe fall on the head. The neck was bent completely to the right side, the upper extremities being paralyzed, attended with hiccup and convulsions. Replacement was immediately attempted, and succeeded. I made the patient sit on the ground, and had the head drawn straight upwards by a strong assistant. The patient got well under the employment of cold locally." Mr. Bell mentions a case of complete dislocation between the last dorsal and first lumbar vertebræ, with entire division of the spinal marrow—but a small portion of bone was broken off.

"The greater mobility (says Mr. Lawrence) of the individual bones, the comparative smallness of their bodies, and the obliquity of the articular processes, point out the cervical vertebræ as those most likely to be luxated; at the same time, the form of the neck, and its connexion with the head, are favourable to the application of such violence as may cause luxation. Hence not only does dislocation of the atlas occasionally occur, but we have also instances of luxated articular processes in the case of the five inferior cervical vertebræ. Baron Boyer even considers that this may happen without external violence, and that the inferior articular process of a cervical vertebra may be carried in front of the superior articular process of the vertebra below it, by a sudden and forcible rotation of the head and neck towards the opposite side. He says that, 'Desault mentioned in his lectures the case of an advocate, who met with this kind of dislocation, by turning his head suddenly round to see who was coming in at a door situated behind his seat. Chopart also shewed us a young man, 24 years old, in whom a similar accident had occurred, in consequence of an extreme rotation of the head, leaving the head permanently inclined upon the left shoulder.'"

Sir Astley Cooper never saw an instance of the dislocation without fracture, but does not deny its possibility. In the anatomical museum of Bartholomew's Hospital, there are some specimens of this accident; but we proceed to Mr. Lawrence's own cases.

*Case* 1. C. B. aged 22, was brought into Bartholomew's Hospital on the 8th January, 1827. He had slipped while carrying a heavy barrel, and fell with the weight resting on the head and upper part of the back. He was instantly deprived of sensibility and motion in the trunk and limbs. In this state he was carried into hospital, all below the neck paralyzed, except the diaphragm. The chest was motionless—pulse weak—body cold—priapism. No irregularity was perceptible in the line of the spinous processes. He lived till the morning of the 12th, viz. better than three days, when he expired, apparently from interruption of the respiratory process.

DISSECTION. No external displacement could be detected in the dead body. But the following examination shewed the nature of the injury.

"After cutting away the muscles from the back of the spine, the cartilaginous surfaces of the superior articular processes of the fifth cervical vertebra came into view: they were exposed in consequence of the inferior processes of the fourth vertebra having been completely dislocated forwards, and remaining fixed in their unnatural position. The yellow ligaments connecting the laminæ of the two vertebræ (ligamenta subflava) were torn through, and the bifid apex of the fourth spinous process lay in close contact with the basis of the fifth. On the front of the column an unusual projection was observed, but the anterior longitudinal ligamentous expansion was entire. The body of the fourth was completely detached from that of the fifth vertebra, the connecting fibro-cartilage being torn through, and the body of the former projecting by its whole depth in front of the latter. In consequence of this displacement, the antero-posterior diameter of the vertebral canal is lessened by about one-third. The section of the bone was not made till some days after death, so that the recent state of the spinal marrow could not be estimated."

The following curious case happened in the practice of Mr. Wigan, and the preparation was shewn to the Society with that of the former case.

Case 2. A child, at the age of five or seven years became affected with an illness supposed to be hydrocephalus, and, after some time, a swelling took place on the side of the neck, containing obviously a fluid, which increased to a considerable size. Pressure on this affected the brain, and produced coma. It was, therefore, supposed that a communication existed between the fluid in the tumour and that in the head. After a long continuance, the tumour disappeared, together with the symptoms of the supposed hydrocephalus. There was no interruption or diminution of sensation or motion at any time, and the child became active and lively. After a time, disease came on in the lumbar vertebræ, attended with bending forwards of the spine, and the formation of a large lumbar abscess, under which the little patient sunk at the age of 12 years.

The head was examined in hot weather, and the brain had become so soft that the changes produced in it by disease could not be ascertained. Mr. Wigan brought Mr. Lawrence the base of the skull, and they were surprised to see a considerable bony prominence standing up in the right side and front of the foramen magnum. This projection was smoothly covered by the dura mater, and proved to be the dentiform process of the second vertebra. After maceration, they found an extensive displacement of the occiput, atlas, and axis, these bones being firmly consolidated in their new relative positions by bony anchylosis of several articulations. The atlas was partially dislocated towards the left; and, at the same time, thrown a little forwards and upwards—hence the right and posterior part of its bony ring intercepted a considerable portion of the spinal canal.

"The middle anterior protuberance now corresponds to the left side of the basilar process; the extremity of the left transverse process projects three quarters of an inch beyond those of the two following vertebræ, while the right transverse processes of those vertebræ project one quarter of an inch beyond the corresponding one of the atlas. A considerable part of the right side of this bone has been destroyed by absorption: that is, the surfaces by which it is articulated to the occiput and atlas, a part of the transverse process, and that groove on which the right vertebral artery rested. The axis is completely dislocated from the atlas and occiput to the right, so that its left portion intercepts about one third of the spinal canal, and the dentiform process projects by its whole length, into the cavity of the skull at the anterior part of the foramen magnum, close to the right anterior condyloid foramen."

The state of parts cannot, indeed, be well understood without seeing the preparation or the drawing; but it is astonishing, says Mr. L. that the immediate pressure of the bony projection on the under surface of the medulla oblongata, caused no paralytic affection—" even when we allow for the very gradual manner in which it must have been produced." Mr. Lawrence will find, on searching the records of pathological facts, that this " gradual manner" of encroachment on vital organs is every thing. We see the brain itself reduced almost to a shell by the pressure of collected water in the ventricles, with scarcely a diminution of the physical or intellectual functions of the sensorium—why then should we wonder that the medulla oblongata accommodated itself to the slow growth of a bony projection in its neighbourhood?

In some interesting observations on diseases of the cervical vertebræ, Mr. Lawrence quotes a passage from a paper of Professor Rust, of Vienna, as published first in a German Journal, and afterwards enlarged in his work on Diseases of the Joints, where he states, that he saw thirteen cases of the complaint.

" Pain in the neck, becoming more severe at night, or in swallowing a large mouthful, or drawing a deep breath, is the first symptom. This pain affects one side of the neck, especially when the head is moved towards the shoulder; it extends from the larynx towards the nape, and often to the scapula of the pained side. No external alteration is perceptible; but firm pressure on the region of the first and second vertebræ produces considerable pain, and thus points out the seat of disease. The difficulty of swallowing and breathing, and hoarseness increase, alternating with pain in the neck, which seems to fix about the back of the head, and becomes intolerable on moving that part. The head sinks towards one shoulder, the face being turned a little down, for in general the articulations are affected on one side only, and that was the left in seven out of nine examinations after death. If both sides are affected the head will incline directly forwards. In this state things continue for several weeks or months; and before worse symptoms come on, there is often apparent improvement, freer motion, and more natural situation of the head. But the uneasiness in speaking and swallowing returns, the pain becomes more severe and extensive, the head falls a little backwards and sinks towards the opposite side. The patient feels as if the head were too heavy, and he carefully supports it with his hands, when he moves from the sitting to the lying position, or vice versâ. This may be considered a pathognomonic symptom of the affection. Another symptom, which at this period shows the true nature of the disease, is a peculiar expression of pain in the countenance, which, combined with the position and stiffness of the head, constitutes so characteristic an assemblage of appearances, that it is enough to have seen it once, in order to recognize it again immediately. This look of the patient, which Rust has endeavoured to represent in an engraving, consists especially in a general alteration of the features, with heavy motion of the eyes, and a dull melancholy expression of internal painful sensations. More active indications of severe suffering are observed whenever the head is moved.

" In the further progress of the case, noise in the head, deafness, giddiness, cramps and convulsions, partial paralysis, particularly of the upper limbs, loss of voice, purulent expectoration, and hectic symptoms supervene. Generally, no external change is observable, either in the neck or in the nape; and Rust observed, in one case only, swelling of the affected side, which broke and left fistulous ulcers. But the slightest pressure in the region of the three upper vertebræ is acutely painful, and sometimes in the advanced period of the disease a grating of rough surfaces is distinctly perceptible when the head is turned. The patient may continue for months in this helpless and painful state, and then dies, either from exhaustion and debility, or, which is more frequent, suddenly and unexpectedly."

Such is Rust's description of a disease which Mr. Lawrence thinks we can have no hesitation in regarding as originally ulceration of the cartilages, proceeding to destruction of the ligaments, and caries of the bones, with extension of disease, in various shapes and degrees, to the neighbouring important parts. Several allusions are made to cases observed by Reil, Bell, and others, of this affection. Rust thinks the complaint must be almost always fatal—at least it proved so in nearly all his own cases.*

In regard to the treatment, it is evident that the principles which are applicable to disease seated in other parts of the vertebral column, ought here to be put in force. On account of the proximity of great and important organs in this last disease, it is a matter of great consequence to lessen the degree and extent of inflammation, and thus limit the disorder in its early stage. Local depletion and the other antiphlogistic plans—perfect quietude—and counter-irritation, are the principal means, by which we can hope to control this formidable complaint, when it is capable of control, or within the range of cure.

In this paper, Mr. Lawrence displays his usual diligent research for materials, especially among German writers. It is not every costive English brain that could spin out thirty-four goodly octavo pages of letter-press, from two cases, one of which was not his own.

The more we see, and hear, and read, of Mr. Lawrence, the more we admire his unwearied research, his acuteness of observation, and his talent for converting the facts of others into plausible supports for his own opinions. He is generally judicious—but hardly ever original. His perceptions of what is going on under his eye appear to be very clear; but we do not think him capable of very extended or enlightened views of disease in general. In fine, we believe him to be an excellent surgeon, but only a second-rate physician. It is no small merit, however, to shine in any one of these two extensive departments of human knowledge.

---

* Although we do not profess to be such "learned Thebans" as the author of this paper, yet we cannot but wonder that the work of Johannes Baptista Paletta,* the first volume of which was published in 1820, should have escaped his vigilant research. The 15th chapter of that work is dedicated to fractures and dislocations of the vertebræ, in which are recorded cases of every description, from the atlas down to the last lumbar vertebra. In the next Essay which Mr. Lawrence favours us with, he may draw on this work for very ample materials indeed.

* *Exercitationes Pathologicæ*, 2 *vols*. 1820—1826. *Milan and Paris*.

